# Surface chemistry and effects on bone regeneration of a novel biomimetic synthetic bone filler

**DOI:** 10.1007/s10856-015-5483-6

**Published:** 2015-03-19

**Authors:** Marco Morra, Gianluca Giavaresi, Maria Sartori, Andrea Ferrari, Annapaola Parrilli, Daniele Bollati, Ruggero Rodriguez Y. Baena, Clara Cassinelli, Milena Fini

**Affiliations:** 1 Nobil Bio Ricerche Srl, Via Valcastellana 26, 14037 Portacomaro, AT Italy; 2 Laboratory of Biocompatibility, Technological Innovations and Advanced Therapies, RIT Department-Rizzoli Orthopaedic Institute, Via di Barbiano 1/10, 40136 Bologna, Italy; 3 Laboratory of Preclinical and Surgical Studies, Rizzoli Orthopaedic Institute, Via di Barbiano 1/10, 40136 Bologna, Italy; 4 Department of Clinical, Surgical, Diagnostic and Pediatric Sciences, School of Dentistry, University of Pavia, Viale Brambilla 74, 27100 Pavia, Italy

## Abstract

The paper presents results of physico-chemical and biological investigations of a surface-engineered synthetic bone filler. Surface analysis confirms that the ceramic phosphate granules present a collagen nanolayer to the surrounding environment. Cell cultures tests show that, in agreement with literature reports, surface-immobilized collagen molecular cues can stimulate progression along the osteogenic pathway of undifferentiated human mesenchymal cells. Finally, in vivo test in a rabbit model of critical bone defects shows statistically significant increase of bone volume and mineral apposition rate between the biomimetic bone filler and collagen-free control. All together, obtained data confirm that biomolecular surface engineering can upgrade the properties of implant device, by promoting more specific and targeted implant-host cells interactions.

## Introduction

To support efficient installation of dental implants after restoration of alveolar bone loss, or sinus lift surgery, or to fill periodontal and periimplant bone defects, many different bone substitutes such as autografts, xenografts, allografts and osteoactive agents have been proposed [1–7]. Among them, xenograft and synthetic bone fillers present the advantage of an unlimited supply of available material and lack of morbidity by eliminating the donor site. Xenografts, in particular from bovine source, take a good share of the market, because the fine microarchitecture and properties of the original bone tissue, together with a good record of clinical performances, make them a reliable alternative to autograft [8, 9]. Among synthetic bone fillers, mostly based on phosphate ceramics, represent the major and widespread bone reconstructive and replacement tool employed in the orthopedic field, because they offer the opportunity of advanced material engineering [10–12]. Structural, chemical and morphological properties (the ratio between different components—mostly hydroxyapatite and β-tricalcium phosphate-, topography, porosity, Ca/P ratio, grain size and shape, effect of atomic substitutions) have been widely investigated [13], in order to maximize the rate of new bone formation and finely tune the degradation rate obtaining “biomimetic” biocompatible materials that closely mimic the inorganic components of bone tissue. Nevertheless synthetic bone graft substitutes currently possess only osteointegrative and osteoconductive properties.

Surface engineering is another tool offered by material science to upgrade properties of synthetic bone fillers. In particular, biomolecular modification of surfaces is an effective strategy to improve device-tissue interaction, by presentation of signaling molecules able to direct cell behavior and stimulate healing at the device-tissue interface [14–16]. In the field of bone-contacting devices, a number of papers highlights the merits of surface modification by type I collagen in periimplant bone regeneration. Collagen type I is the most abundant protein in bone, where it makes up approximately 85 % of the organic portion. Cellular interactions with collagen have been shown to be important in the regulation of the osteoblast phenotype. Collagen controls adhesion of cells of direct relevance to bone-contacting applications [17], through the amino acid sequence Arg-Gly-Asp (RGD) that it contains. It plays an important role in osteoblast cells behaviour, promoting not only cell adhesion, but also osteoblastic differentiation of bone marrow cells and controlling a number of aspects of their progression along the osteogenic pathway [18–20]. RGD interacts with critical biomolecules and growth factors, providing the cooperative signaling required for BMPs functioning [21]. It exerts a strong pro-coagulant (hemostatic) activity and it activates platelets in a unique way [22]. This aspect is of definite interest on the light of the role of early blood–implant interactions in bone healing [23–25]. For the reasons mentioned above, collagen is commonly used in dental and orthopedic surgery as osteogenic and bone filling material, where it provides a more rapid regeneration of bone defects. Even some xenograft filler takes advantage of collagen properties, by low temperature processing that keeps collagen within the inorganic bone matrix.

Recently, a new synthetic biomimetic bone filler that exploits biomolecular surface engineering to deliver directly to the filler-implant interface the signaling properties of type I collagen has been developed. Contrary to other fillers where it is part of the bulk of the material or a major component of the formulation, in the present case collagen is just contained as a surface cross-linked nanolayer, whose thickness is of the order of nanometers, on top of 25 % hydroxylapatite, 75 % β-tricalciumphospate granules. It is expected that interfacial interaction between host cell and the surface nanolayer can stimulate bone regeneration mechanisms, promoting faster and more consistent bone formation in the defect. Thus, despite being synthetic in nature, this new synthetic biomimetic bone filler can play an active biological role in the healing process, by collagen-host cells communication.

To the authors’ knowledge, the biomolecular surface engineering applied to synthetic calcium phosphate fillers represents an innovative approach to upgrade materials properties that requires in vitro and in vivo preclinical investigations. Thus the scope of this paper was to investigate the specific surface chemistry and relevant biological implications of this newly developed synthetic bone filler. To this end, the surface chemical composition of tested bone filler was evaluated by techniques that probe different sampling depths, such as attenuated total reflection infrared spectroscopy (ATR-IR) and X-ray photoelectron spectroscopy (XPS). Human mesenchymal cells cultures on tested bone filler and relevant control were used to evaluate in vitro, through RT-PCR, the expression of the gene encoding for alkaline phosphatase, the main marker of progression along the osteogenic pathway. Finally, bone regeneration in vivo was investigated in a rabbit model of bone defect.

## Materials and methods

The following samples were tested:

1. Synthetic bone filler (SB) based on 25 % hydroxylapatite (HA)–75 % β-tricalciumphospate (β-TCP) granules, 0.3–1 mm size range, bearing a surface cross-linked nanolayer of collagen form porcine source
2. Collagen-free, 25 % hydroxylapatite–75 % β-tricalciumphospate control granules (SB-), 0.3–1 mm size range.

Samples were prepared as follows:

A slurry obtained by thorough mixing of 75 % β-TCP (Sigma) and 25 % HA (Fuildinova, Portugal) in a 3 % aqueous solution of guar gum was prepared. Disks, about 4 cm diameter, were dried at 100° C in a oven. Dried disks were pressed for 10 s at 170 bar using a Mignon EA/SSN press. Disks were then sintered at 1100 °C, with a 1 h dwelling time and a 1 °C/min ramp rate, using a Lenton ECF 12/4 chamber furnace.

Sintered samples were then mechanically grinded and the 0.3–1 mm fraction recovered through calibrated analytical sieves (Retchs). Complete characterization of the bulk phases of the obtained material is reported elsewhere (Morra M. et al., submitted manuscripts).

Half of the obtained material was packaged in sterile vials and coded SB-. The remaining half was subjected to the collagen coating process. Shortly, granules were placed in a 0.1 % collagen type I solution (TheraCol, Sewon Cellontech Co., Ltd., Korea). After overnight incubation at room temperature the collagen solution was then displaced by phosphate buffer and samples rinsed with pyrogen-free MilliQ water. Cross-linking of the adsorbed collagen layer was obtained by adding water-soluble carbodiimide (EDC) and N-hidroxysuccinimide (NHS) to the aqueous solution containing the samples. After 2 h, samples were washed and dried under a hood (SB). Further to packaging both SB and SB- were gamma sterilized.

### Surface analysis investigations

#### ATR-IR

ATR-IR spectra were obtained using a Nicolet iS10 ATR-IR spectrometer, produced by Thermo Scientific and equipped by a diamond crystal. SB and SB- granules were gently placed on the crystal and kept in place by the specific crimping tool. The experimental set up involves acquisition of 32 scans in the range 500–4000 cm^−1^, both of sample and background, at a resolution of 4 cm^−1^ [21].

#### XPS

XPS analysis was performed with a Perkin Elmer PHI 5400 ESCA system. The instrument is equipped with a Mg anode operating at 10 kV and 200 W. The diameter of the analyzed spot is approximately 2 mm, the base pressure 10^−8^ Pa. The angle between the electron analyzer and the sample surface was 45°.

Measurements were performed by pressing SB and SB- granules, in order to make a complete layer on a double sided adhesive tape, one side of which was fixed to the instrument sample holder.

Analysis was performed by acquiring wide range survey spectra (0–1000 eV binding energy) and detailed high resolution peaks of relevant elements.

Quantification of elements was accomplished using the software and sensitivity factors supplied by the manufacturer.

### Human mesenchymal stem cell (hMSC) culture

hMSC from bone marrow were purchased from Cambrex Bio Science (Milan, Italy). On arrival, the culture medium was immediately removed and replaced by new Mesenchymal Stem Cell Basal Medium (MSCBM, Cambrex Bio Science) supplemented with 10 % fetal bovine serum and l-glutamine, penicillin/streptomycin (all from Cambrex Bio Science).

At subconfluence, cells were harvested by adding sufficient volume of Clonetics Trypsin–EDTA (CC-3232, Milan, Italy) solution to cover the cell layer, and gently rocking the flask. After blocking Trypsin by serum, cells were seeded in the experimental wells (described below) at a density of about 7 × 10^4^ cell/mL, as evaluated by TC10 automated cell counter (Bio Rad, Milan, Italy).

For the experiments a thin layer of SB and SB- (about 0.20 g) was separately spread on the bottom of 24 multiwell plates (Falcon, Becton&Dickinson, Milan, Italy), completely masking the underlying plastic substrate. Three replicate wells were used for each experimental time. Cells were seeded on wells containing SB or SB- and cultured at 37 °C in a humidified incubator equilibrated with 5 % CO_2_, in the following culture media:

- Ostegenic medium: the cell culture medium was supplemented with hMSC differentiation bullet kit (PT-3002, Cambrex Bio Science), which contains dexamethasone, ascorbate, l-glutamine, penicillin/streptomycin, β-glycerophosphate;
- Non differentiating medium: culture medium without osteogenic differentiation supplement factors.

### RT-PCR

The expression of ALP gene as a cell differentiation markers was assessed using the real time reverse transcription polymerase chain reaction (qRT-PCR). hMSC cells were cultured on the described materials and total RNA was extracted at 3, 7 and 14 days time points using MagMax Total RNA Isolation Kit (Applied Biosystems, Milan, Italy) following the manufacturer’s instructions. RNA quality was assessed by checking the A260/A280 ratio (1.6–2.0). Then total RNA was used as a template for cDNA synthesis using random hexamers as primer and multiscribe reverse transcriptase (high capacity cDNA RT Kit from Applied Biosystems).

cDNA amplification and relative gene quantification was performed using Taq Man probe and primers from Applied Biosystems (Hs 01029144_m1, ALP). Real time PCR was performed in duplicate for all samples and targets on a Step-One instrument (Applied Biosystems) using the software Step-One, version 2.1. PCRs were carried out in a total volume of 20 μl and the amplification was performed as follows: after an initial denaturation at 95 °C for 10 min, the PCR was run for 40 cycles at 95 °C for 15 s and at 60 °C for 1 min.

To normalize the content of cDNA samples, the comparative threshold (Ct) cycle method, consisting on the normalization of the number of target gene copies versus the endogenous reference gene GAPDH, was used. The Ct is defined as the fractional cycle number at which the fluorescence generated by cleavage of the probe passes a fixed threshold baseline when amplification of the PCR product is first detected. For comparative analysis of gene expression, data were obtained using the ΔCt method.

#### Determination of in vivo histocompatibility

##### Animal model

After synthetic bone filler characterization, in vivo evaluations have been performed also following the international rules UNI EN ISO 10993-6: 2009 “Biological evaluation of medical devices—tests for local effects after implantation” in order to obtain information about material in vivo performance in terms of histocompatibility and osteointegrative capabilities. All surgical care and procedure were performed following the Italian and European Laws on animal experimentation. The research protocol was approved by the Ethical Committees of the Rizzoli Orthopaedic Institute and by the public authorities as provided by Law by Decree 116/92.

Ten male New Zealand rabbits (HARLAN Laboratories SRL, S.Pietro al Natisone—Udine), 3.06 ± 0.15 kg body weight, were housed in individual cages and fed with a standard pellet diet (Mucedola, Settimo Milanese, Milano, Italia) and water ad libitum used. After a quarantine period of 10 days, the animals were submitted to surgery for experimental materials placement in the trabecular bone tissue of femoral condyles. The animals were intramuscular injected with 44 mg/kg ketamine (Imalgene 1000, Merial Italia S.p.A, Assago-Milan, Italy) and 3 mg/kg xylazine (Rompun, Bayer SpA, Milano, Italy) were performed to induce general anesthesia. During surgery anesthesia was maintained with O_2_/air (1/0.4 l/min) mixture and 2–2.5 % sevorane (Sevorane, ABBOTT Srl, Latina, Italy).

Longitudinal incisions on the lateral surface of femoral condyles were performed bilaterally and distal femurs exposed. Critical size defects of 6 mm in diameter and 10 mm in depth were created with a low speed drill, flushing and cooling with sterile 0.9 % NaCl to remove bone debris. Subsequently, SB and SB- materials were implanted in left and right femoral condyles, respectively, and wounds were sutured in layers. Postoperatively, antibiotics and analgesics therapies were administered. To monitor the dynamic of bone ingrowth, the animals received i.m. injections of oxytetracycline fluorochrome (30 mg/kg b.w., Terramicina 100, Pfizer Italia, Italy) 2 days on, 10 days off and 2 days on, 18 days before the end of experimental time.

Twelve weeks after surgery, animals were sacrificed with the intravenous administration of 1 ml of Tanax (Tanax, Hoechst, Frankfurt am Main, Germany) under general anesthesia as previously reported. Femoral condyles were explanted and during dissection, the presence of clinical signs of inflammation, haematomas, oedema, or tissue reactions were macroscopically evaluated; also lymphatic draining district (popliteal and inguinal lymph nodes) was taken into consideration.

##### Microtomographical, histological and dynamic histomorphometric evaluations

Microtomographic assessment was carried out onto femoral condyles samples using the Skyscan 1172 computed microtomographic system. The scans were performed with a source voltage of 100 kV, at a source of 100 μA and using an aluminum filter 0.5 mm. Each sample was rotated until 180° with a rotation step of 0.4° and a frame averaging of 4. The pixel size was 13.5 μm. The reconstructions were performed using NRecon (v1.6.2.0) software and the resulted jpg images had 2000 × 2000 pixels with a pixel size of 13.5 μm. For reconstruction the specific misalignment for each acquisition, a small smoothing and a beam hardening correction of 20 % were applied.

The reconstructed crossectional images constitute the whole volume for each sample. Using the CTAn software (Skyscan, Belgium) the datasets of these images were elaborated to perform the 3D analysis (directly on the volume). A cylindrical volume of interest (VOI) corresponding to the surgical defect (6-mm in diameter and 10-mm in height) was considered for each sample. Within the VOI the following parameters were calculated:

- Volume of the material in the defect (Mat.V/TV, %): expressed as a ratio between the volume of the material in the VOI and the total volume of the considered VOI;
- Bone volume density (BV/TV, %): expressed as a ratio between the volume of newly formed bone measured in the VOI and the total volume of the considered VOI;
- Ratio between bone tissue surface and measured tissue volume (BS/BV, mm^−1^): expressed as a ratio between the bone tissue surface inside the VOI (BS, mm^2^) and the volume of the newly formed bone inside the VOI (BV, mm^3^).

After micro-CT scan, femoral condyles were processed for non-decalcified histology. Bone sample were immediately fixed in 4 % paraformaldehyde, dehydrated in an ascending series of ethanol concentrations until the absolute one and finally embedded in a methyl methacrylate resin solution (Methyl Methacrylate stabilized for synthesis, Merck Schuchardt OHG, Hohenbrunn, Germany). Serial transversal sections were obtained by using rotative microtome (Leica SP1600, Leica Microsystems Spa, Milan) with a 40 ± 10 μm thicknesses. The obtained sections were then ground and polished until 15 ± 5 μm thicknesses with Saphir system (Saphir 550, ATM GmbH, Mammelzen, Germany) and left unstained for dynamic histomorphometry or stained with Stevenel Blue counterstained with Picrofucsin according to Van Gieson method for qualitative histological evaluations.

To perform dynamic histomorphometric investigations, semi-automatically light/fluorescence microscope (BX51, Olympus Optical Co. Europe GmbH, Germany) connected to an image analyzer system (Qwin, Leica Imaging Systems Ltd., United Kingdom) was used and different regions of interest (ROIs) were grabbed at 20× magnification. The following histomorphometric parameters were evaluated:

- Mineral apposition rate (MAR, μm/day): measured as the distance between the midpoints of two consecutive deposited and epifluorescent fronts of fluorochrome divided by the time between the the labelling period.
- Bone formation rate (BFR/B.Pm μm2/μm/day): BFR/B.Pm values were obtained by multiplying the MAR value by the sum of ½ single label perimeter (sL.Pm) and double label (dL.Pm) perimeter as explained by the following formula:

$$MAR*(1/2 \, sL.Pm/B.Pm + dL.Pm/B.Pm)$$

All stained histological sections were finally digitized adopting a digital scanner at full resolution (1781 × 1467 pixels).

##### Statistical analysis

Statistical analysis was performed using SPSS v.12.1 software (SPSS Inc., Chicago, Illinois). Data are reported as mean ± standard deviation (SD) at a significance level of *P* < 0.05. After having verified the normal distribution and the homogeneity of the variance, Student *t* test for paired data was used, to assess the significant differences between the SB and SB- materials.

## Results

### Surface analysis

**Fig. 1:**
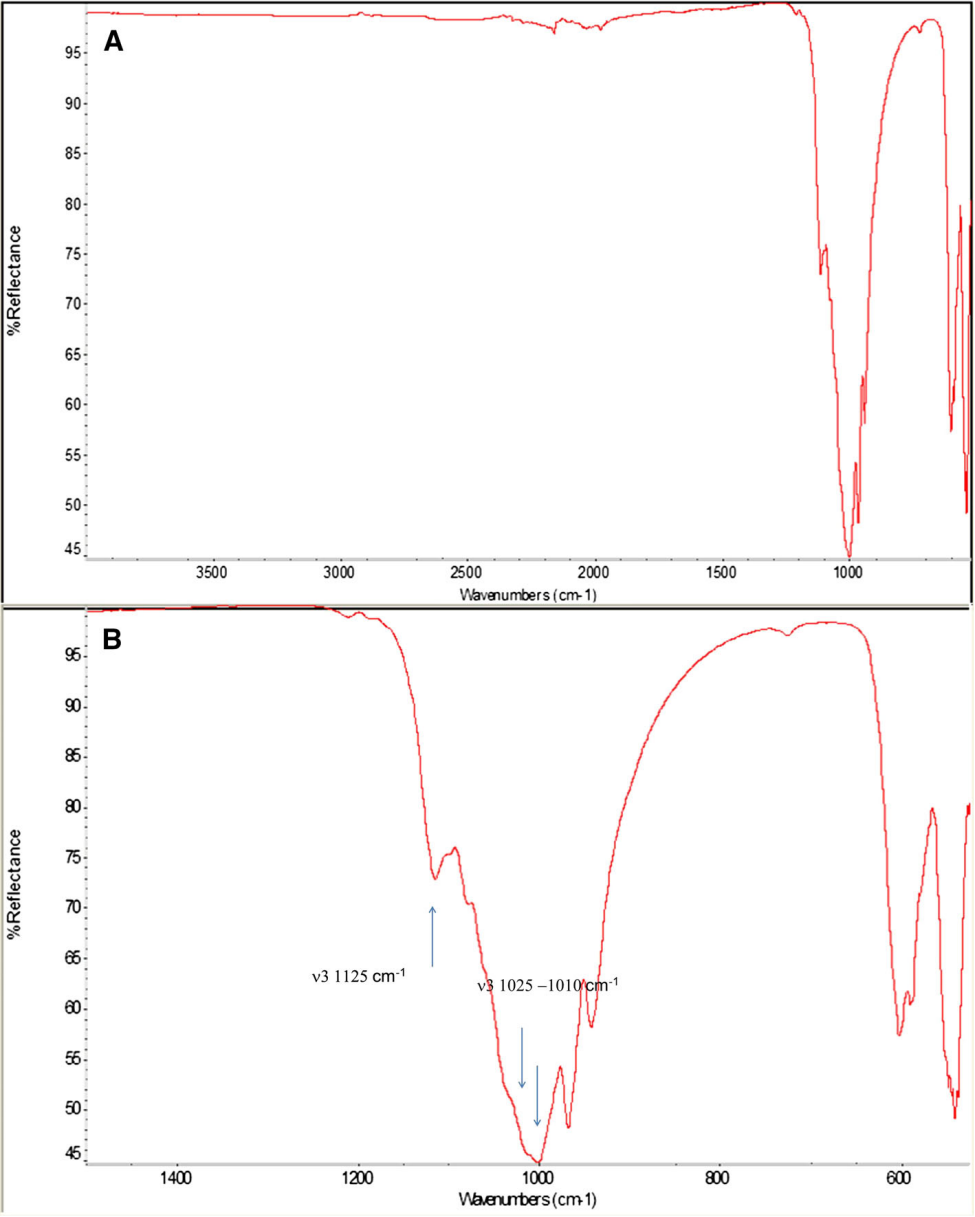
**a** ATR-IR spectrum of SB in the range 500–4000 cm^−1^; **b** detail of triply degenerated asymmetric stretching mode (ν3) of the P–O bond of the phospate group

ATR-IR spectrum of SB in the range 500–4000 cm^−1^, is shown in Fig. 1a. The most important observation from this spectrum is that only peaks belonging to the inorganic phase, that is the intense phosphate stretching bands observed at around 1010 and 560 cm^−1^, were detected. The phosphate band is reported in more details in Fig. 1b. Confirming the claimed composition, it contained features belonging both to hydroxylapatite and β-tricalciumphospate (950 and 1140 cm^−1^) [26]. The main features of the triply degenerated asymmetric stretching mode (ν3) of the P–O bond of the phospate group is shown in Fig. 1b. The 1125 cm^−1^ peak is typical of tricalciumphospate, while peaks at lower wavenumber bear features both of tricalciumphospate and hydroxylapatite [27].

In this respect, the SB spectrum was definitely identical to that of SB- (not shown). No evidence of the presence of collagen was gathered from this analysis; proteinaceous compounds should show the typical amide peaks in the 1500–1700 cm^−1^ range.

**Fig. 2:**
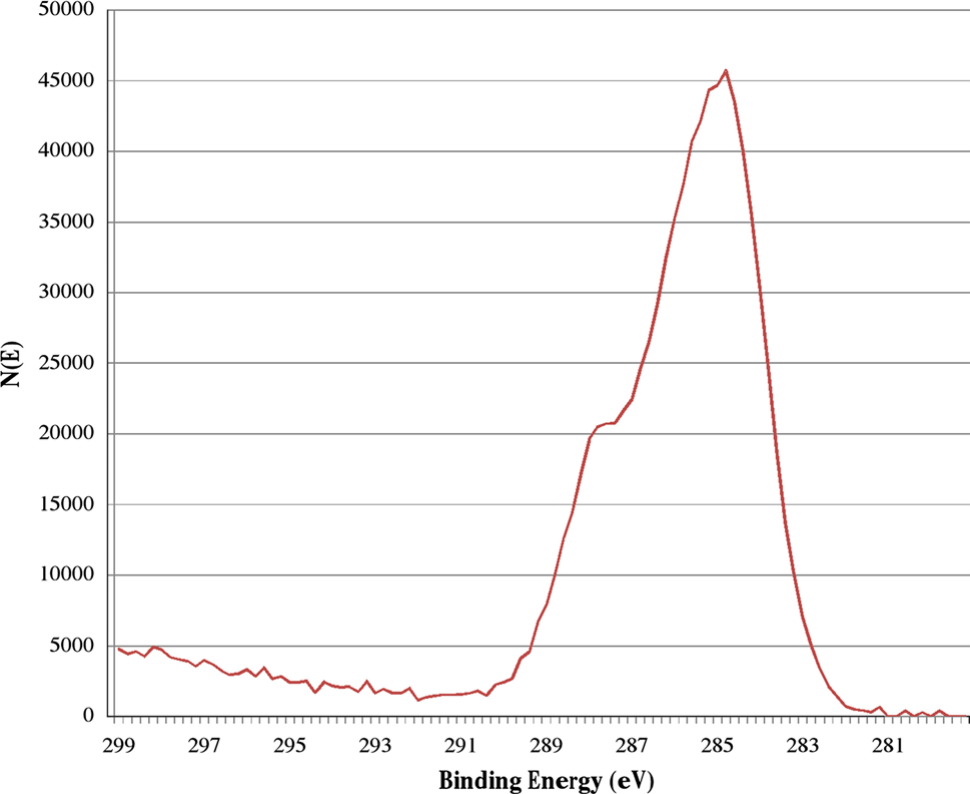
High resolution C1 s peak of SB

The chemical composition of the surface of SB and SB-granules, as detected by XPS analysis, is reported in Table 1. Data show a very significant difference between elemental distributions on the granule surface. SB-, as expected, contained a high percentage of Ca and P, and no N. SB, on the other hands, showed a significant amount of nitrogen, a marked reduction of Ca and P, and an increase of the carbon content. The overall stoichiometry detected on SB was consistent with that of collagen and of collagen coated implants [27]. Further indications were gathered from the high-resolution C1 s peak of SB (Fig. 2). The peak showed the typical shoulder at about 288–289 eV, that is 3.5–4 eV shift from the main C–C, C–H component, due to C in the amide bond chemical environment.

**Table 1:** Surface composition, as detected by XPS, of SB- and SB granules surface composition (at.%)

Sample	O	Ca	C	P	N
SB-	45.8	21.9	20.8	11.6	
SB	19.3	5.2	61.9	1.7	11.9

### hMSC culture

hMSC were cultured on SB- and SB layers, both in osteogenic and non-differentiating media. The main hallmark of progression of undifferentiated hMSC along the osteogenic pathway was the increase of ALP expression.

**Fig. 3:**
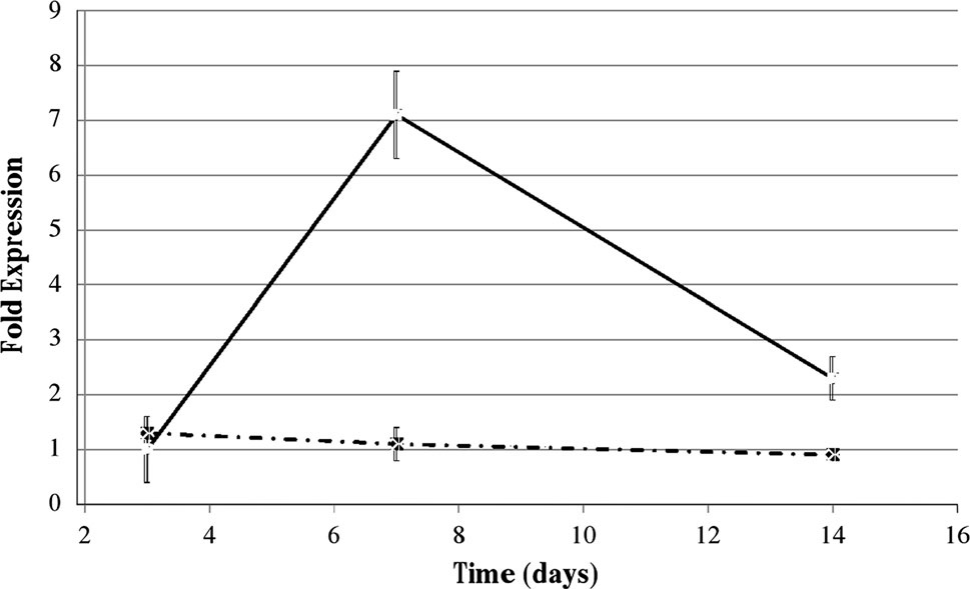
Fold expression of ALP gene by hMSC cultured on SB over hMSC cultured on SB-. *Diamond*: osteogenic medium; *filled square*: non differentiating medium. Differences are statistically significant (*P* > 0.01) for 7 and 14 days datapoints

Results of RT-PCR measurement of ALP gene expression are shown in Fig. 3. Measurements were performed after 3, 7 and 14 days culturing. Data are reported as fold expression of ALP gene expression by hMSC cultured on SB over ALP gene expression by hMSC cultured on SB-. Considering first data obtained in osteogenic medium, fold expression is always close to one, meaning that ALP gene expression is almost identical between cells cultured on SB- or SB. Interestingly, data obtained in non-differentiating medium show a completely different trend: while fold expression is close to one after 3 days, induction time, very significant overexpression of ALP gene is detected after 7 and 14 days for cell cultured on SB.

#### Determination of in vivo histocompatibility

Anesthesia procedures and postoperative healing period were uneventful for all animals that fulfilled the predetermined experimental time without complications, except for an animal that was excluded from ex vivo assessments due to the occurrence of a microfracture at the implant site in which the experimental material was implanted (in the absence of clinical signs). Surgical defects healed without complications and macroscopically no adverse tissue bone tissue or periimplat tissue reactions occurred. The observation of popliteal and inguinal lymph nodes showed no signs of swelling or morphological alteration due to inflammatory or degenerative processes.

##### Microtomographic and dynamic histomorphometric evaluations

**Fig. 4:**
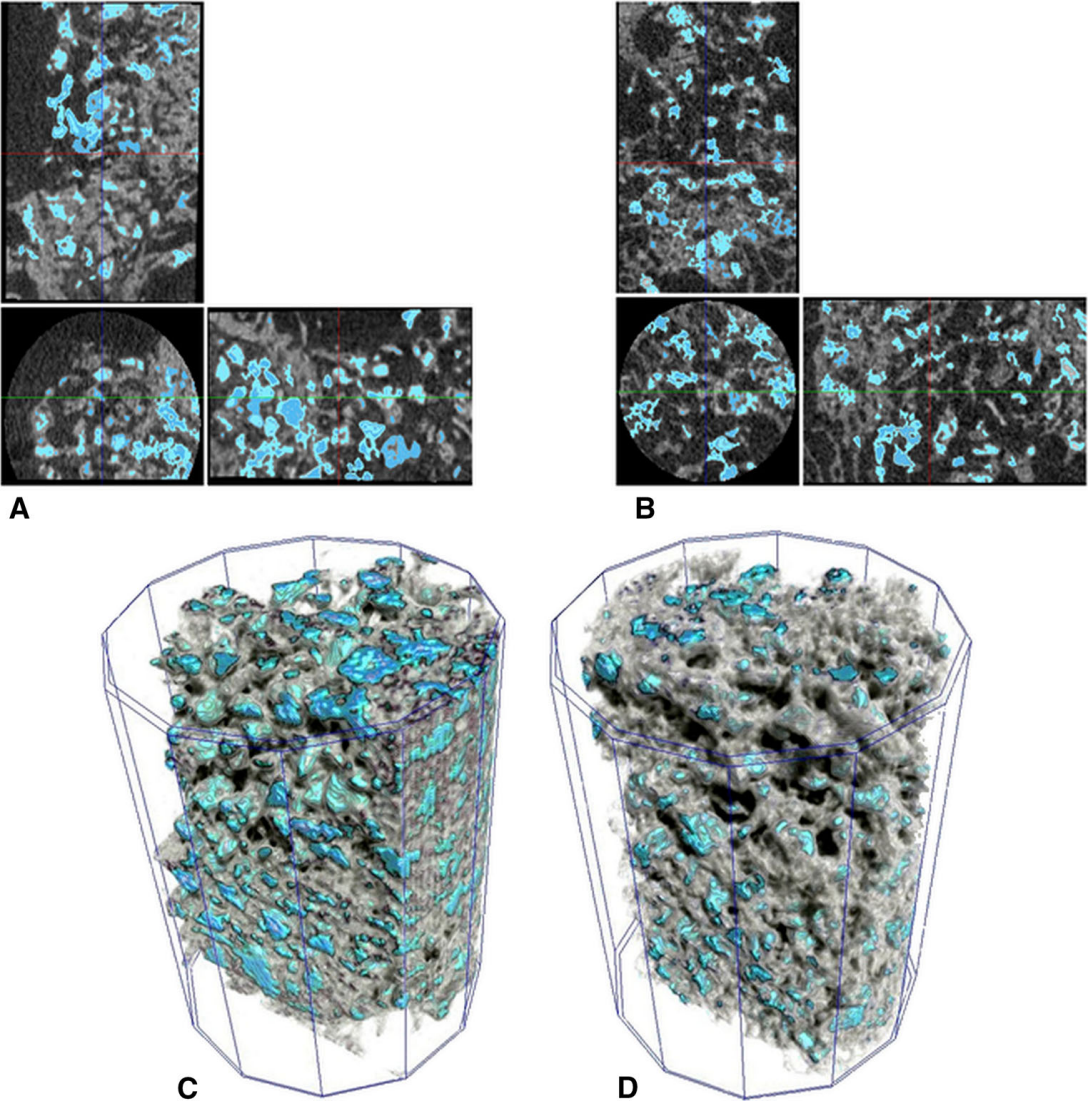
Volume of interest (VOI) selected to perform the micro-CT measurements, according to the three scanning planes: longitudinal, coronal, and sagittal respectively of SB (**a**) and SB- (**b**) material. Tridimensional reconstruction of the volume of interest (VOI) selected to perform the microtomographic investigations of SB (**c**) and SB- (**d**) materials. The *blue color* areas correspond to the materials, while those in *dark gray* correspond to the newly formed bone tissue (Color figure online)

**Fig. 5:**
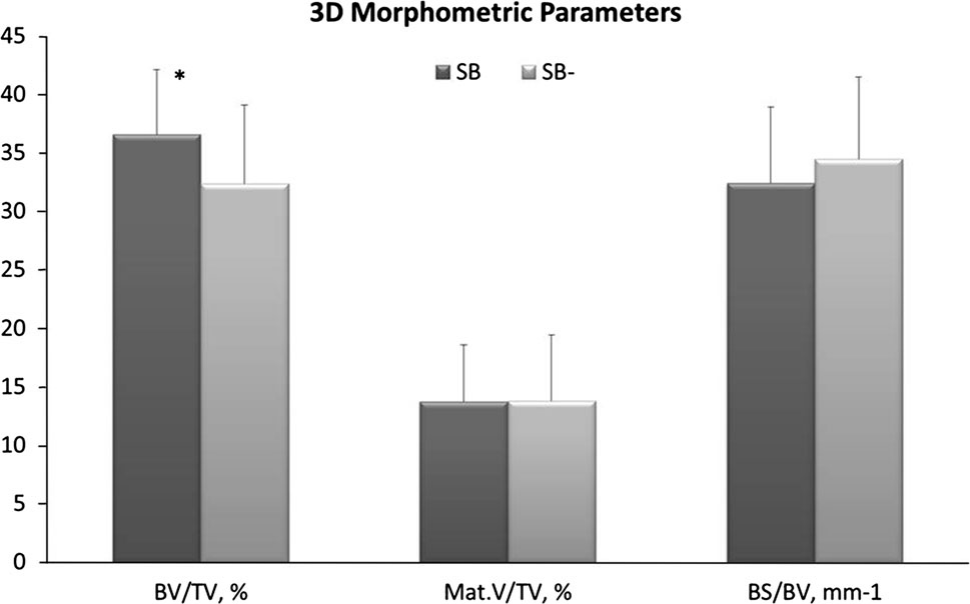
Histograms of 3D morphometric results (BV/TV, %; Mat.V/TV, %; BS/BV, mm-1) obtained for SB and SB- materials at 3 months (mean ± SD; n = 9 implant sites). Student *t* test: *P < 0.05

The VOI selected to perform the micro-CT measurements according to the three scanning planes (longitudinal, coronal, and sagittal) and a tridimensional reconstruction of this were reported in Fig. 4. The statistical analysis onto 3D morphometric results highlighted a significant (*P* < 0.05) difference between SB and SB- materials for bone volume density (BV/TV) as showed in Fig. 5. No significant differences were found between SB and SB- materials for the quantitative evaluation of the material volume (Mat.V/TV) and bone tissue surface (BS/BV) parameters.

**Fig. 6:**
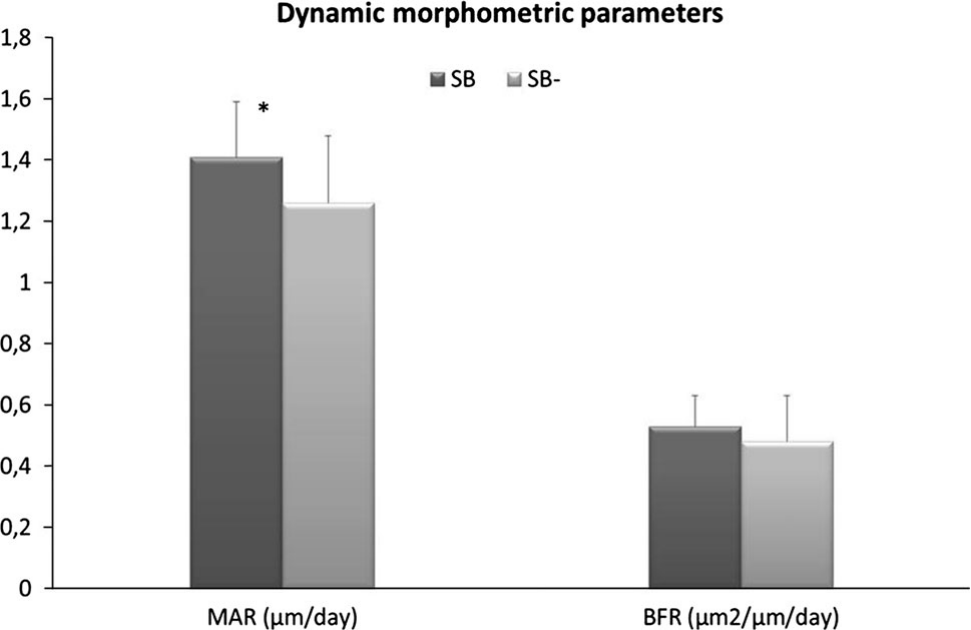
Histograms of mineral apposition rate (MAR, μm/day) and bone formation rate (BFR, m2/μm/day) obtained for SB and SB- materials at 3 months (mean ± SD; n = 9 implant sites)

Concerning the quantitative evaluation of Oxytetracycline fluorochrome, the results were reported in Fig. 6. MAR parameter was statically higher for SB materials respect to SB- (*P* < 0.05), while no significant differences were detected in BFR values between materials.

##### Qualitative histological evaluation

**Fig. 7:**
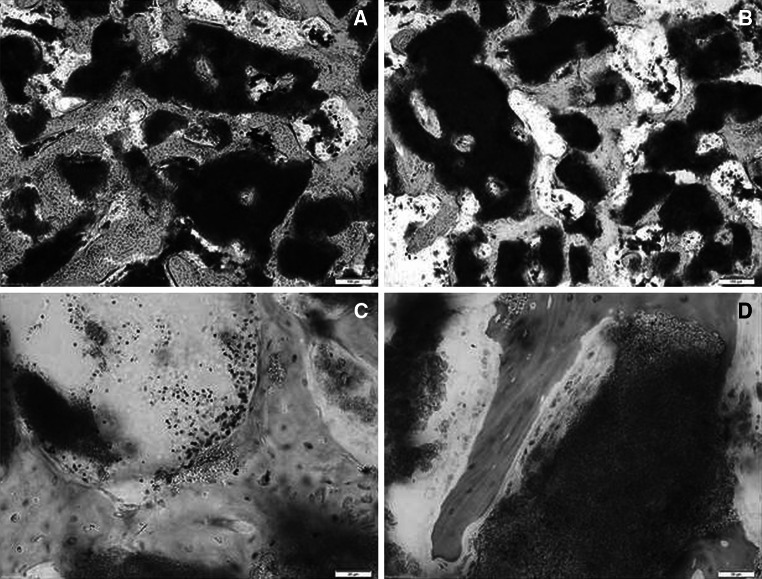
Representative histological images obtained from SB (**a**, **c**) and SB- (**b**, **d**) materials 3 months after surgery. Stevenel *Blue* staining counterstained with Picrofucsin according to Van Gieson was performed. (*scale bar* **a** and **b** = 100 μm; **c** and **d** = 20 μm) Materials granules of both materials (**a**, **b**) were almost completely surrounded by bone tissue, which grows tight in contact with the surface of them without the interposition of fibrotic or inflammatory tissue. Granules materials appeared as “embedded” in bone tissue that grew forming bridges between granules of well-structured trabeculae. Active areas of deposition were detectable in close proximity of both materials (**c**, **d**): osteoid was extensively deposited along the surface of bone trabeculae and above them rows of osteoblasts were aligned (Color figure online)

Neither inflammation nor adverse tissue reaction were detected around both granules materials and no immunogenic event triggered by the presence of bovine collagen occurred. As expected, 3 months after surgery, the created defects were completely occupied by newly grown trabecular tissue in both SB and SB- material. The histological evaluation revealed no qualitative differences between materials, showing both materials granules almost completely surrounded by bone tissue, which grows tight in contact with the surface of them without the interposition of fibrotic or inflammatory tissue. Granules appeared confined in the previously created surgical defect, but at the same time well distributed, thus avoiding excessive packing or dispersion phenomena (Fig. 7a, b). Cross sectional histologies showed different size and dimension of granules materials that appear as “embedded” in bone tissue that grew forming a bridges between granules of well-structured trabeculae that displayed a similar morphology that closely resembles that of host tissue (Fig. 7a, b). As shown in Fig. 7c, d active areas of deposition were detectable in close proximity of materials: osteoid was extensively deposited along the surface of bone trabeculae and above them rows of osteoblasts were aligned.

## Discussion

Biomolecular modification of implant materials is a promising approach to upgrade properties of conventional devices. In fact, in the last years it was used to functionalize biomaterials by using bioactive molecules such as protein-like collagen, bone morphogenetic proteins, growth factors and peptides and/or protein domain [28–31]. As well known, the host response to a biomedical implant starts with proteins absorption onto materials surface that trigger and dictate the subsequent biological cascade of events [32, 33]. Thus the localization at the implant-host tissue interface of specific signaling molecules can improve implant biological performance and the host acceptance, directing simultaneously the healing, regeneration mechanisms and integration processes.

In this perspective, the surface of calcium phosphate material was bioactive engineered through its cross-linked functionalization with collagen type I nanolayer, to create specific sites of interaction able to elicit osteoblasts and mesenchymal stem cells recruitment, differentiation and proliferation. In fact convincing evidence exists that shows that a surface layer of collagen on titanium fixtures stimulates periimplant bone regeneration [34–44].

Therefore in the present research, a well-defined biphasic ceramic bone filler, as described in the seminal work by Daculsi ([45] and references therein), composed of 25 % hydroxylapatite–75 % β-tricalciumphospate, was further surface engineered with a cross-linked nanolayer of type I collagen from porcine source (SB). The interaction of osteoblasts with uncrosslinked collagen I coating on calcium phosphate ceramic was described by Brodie and coworkers [46]. In the present case the surface-adsorbed collagen layer was further crosslinked, to prevent early disruption of the collagen layer by collagenase in the wound site (a discussion on the role of cross-linking in the stability of collagen surface layers has been presented elsewhere [47]. De Jong and coworkers presented interesting results on osteogenic effect of electrosprayed nanoscale collagen/calcium phosphate coatings. The deposition technique adopted by De Jong and coworkers yields coating thickness of 90 or 120 nm in the quoted paper. In the present study the process in based on protein interfacial adsorption and thinner layers are obtained [47].

Several investigations techniques were adopted to confirm the surface functionalization: ATR-IR and XPS were used to characterize the chemical nature of material surface and the in vitro expression of marker of early osteogenic activity (ALP) was assessed from no-committed mesenchymal cells by RT-PCR. Material histocompatibility was also evaluated in an in vivo model, adopting microtomographic, histological and histomorphometric investigations.

The surface localization of the collagen layer is confirmed by the results of surface characterization. ATR-IR analysis failed to detect contribution from organic phases to the SB spectrum, while XPS clearly detected the turning of surface chemistry from the inorganic phosphate ceramic SB- to the organic, proteinaceous environment SB. In particular, ATR-IR probed a sampling depth slightly below 1 micrometer, but failed to detect the presence of collagen on the inorganic phosphate ceramic matrix (Fig. 1a). This is indeed the case for fillers that contain collagen or proteins in their bulk composition, such as natural bone and the low-temperature treated bovine bone OsteoBiol [48]. On the other hand, XPS analysis, that samples a depth about one hundred times thinner, of a few nanometers, clearly identifies the surface collagen layer (Table 1). These results should be compared with the interesting findings presented by Figuereido and coworkers [48]. In their paper, collagen was detected by ATR-IR of samples where it is a structural component, such as natural bone and the low temperature processed Osteobiol xenograft filler. In those cases, collagen is evenly distributed in the inorganic matrix, while in the present one it is localized at the surface, within a thickness of the order of nanometers. Thus, the picture that arises from present surface analysis data is that of phosphate ceramic granules interact with the surrounding tissue by means of a few nanometer coat of specific collagen type I chemistry.

Results of the evaluation of ALP gene expression confirms that the surface nanolayer successfully provides cues that stimulate progression of hMSC along the osteogenic pathway [20]. This was verified in further separated experiments evaluating also a panel of osteogenesis-related genes (*data not shown*). Current tests performed in non-differentiating medium, without any contribution from soluble stimuli, showed statistically significant differences in ALP expression by hMSC cultured on SB versus SB-. From a cellular standpoint, here SB provided also osseoinduction, or the ability to induce differentiation of pluripotential cells from surrounding tissue to an osteoblastic phenotype. The obvious interpretation is that in both cases the stimuli contained in the culture medium (β-glycerophosphate, ascorbic acid and dexamethasone) promoted progression along the osteogenic pathway of cultured hMSC, over and above any contribution from the culture substrate [20]. The long term (i.e. 14 days) data were probably affected by the onset of cellular degradation in the culture well, and they suggest that the only variable involved in the experiment (the different surface chemistry), can stimulate ALP expression by cultured undifferentiated hMSC. In agreement with reports on the effect of surface layer of collagen on plastic and titanium [20, 49], it could be stated that the surface cross-linked collagen on SB granules offered molecular cues that promoted progression of hMSC along the osteogenic pathway.

Concerning the in vivo evaluation of histocompatibility, the current results proved that SB and SB- bone fillers are both histocompatible and osteoconductive materials as suggested by the formation of new trabecular bone tissue in all surgical defects. Nevertheless, statistical analysis highlighted that a significant difference exists in the amount of mineralized bone tissue (BV/TV) between animals treated with SB compared to those that have received SB- materials (*P* < 0.05). The fluorochrome agent used to quantify and visualize the velocity and the direction of bone tissue growth, revealed a major degree of mineralization for SB material (*P* < 0.05) in comparison with SB- confirming the BV/TV results and suggesting a greater and faster osteoblast activity around granules material.

Histological evaluation revealed no differences in the quality of bone between the engineered surface material (SB) and the control (SB-). A direct apposition of new bone onto both granular surface materials was detected without the presence of inflammatory or adverse tissue reaction eventually evoked by the potential immunogenicity of bovine collagen. Bone tissue appears well organized around both materials granules, which remained confined to the surgical defect area, with an evenly distribution. Thus the drawback frequently reported and related to the excessive packing of granular material was avoided. In fact, as clearly demonstrate by the histological images, bone tissue infiltrated both materials taking advantage by the whole exposed surface.

Several reports in literature, as those published by Stadlinger research group, deeply investigated the effect exerted by material surface coating with organic components of extracellular matrix onto in vivo osseointegration processes [44, 50–52]. In particular, collagen type I, collagen type III, RGD peptides represent the biological molecules most commonly employed to increase materials performance. Also the present authors investigated and confirmed the aforementioned phenomenon in previous published papers, both in vitro and in vivo [39, 43, 53], but to the author’s knowledge no data are yet available onto calcium phosphate synthetic bone fillers engineered with collagen nanolayer.

## Conclusions

Based on the present results, it can be argued that engineered surface functionalized with a collagen type I nanolayer enhance the inherent osteoconductivity properties of calcium phosphate material as demonstrate by the histomorphometric results (BV/TV and MAR parameters). Moreover ALP gene expression by un-differentiated hMSC gives the successful evidence that osteoinductive characteristic was conferred to material by the engineered surface thus overcoming the main disadvantage of calcium phosphate. Finally, the present results confirming that material interaction with the biological environment was facilitated by the exposure of molecule cues that resembles the surrounding physiological extracellular matrix. Simultaneously, they provide firmer site of recognition and adhesion for cells, creating an advantage for the materials, because uncontrolled non-specific protein adsorption was avoided.
